# Luck

**Published:** 1871-01

**Authors:** 


### LUCK.

The man who marries the prettiest girl of the place is said to be a "lucky fellow," and so of him who draws the highest prize in a lottery, or, by some "fortunate" turn in affairs, clears the gulf between want and wealth in an hour. And yet the histories of all times tell us that with a terrible uniformity and certainty the men who become suddenly possessed of unearned millions die in misery.

"Before God I swear," said a young man in our hearing the other day, as with raised hand and upturned eye, so beseechingly, "give me a dollar, and I will spend it right." His father, well known to every New-Yorker, made a large fortune in Broadway, and died a few years ago; and this his son was regarded as a "lucky dog," and now was piteously begging his father's old friend and lawyer for a pitiful dollar, in tones so abject as to compel contempt.

Within five years a well-to-do farmer drew a quarter of a million of dollars in a prize in a lottery. The whole country envied him his good luck; but he has since died from a style of living induced by his good fortune, and his only son has turned out a drunkard.

The man whose first bet on the race-course, whose first deal at the card-table, whose first risk at faro, whose maiden lottery ticket, brings money largely into his pocket, is a ruined man at the very instant the world pronounces him "lucky."

Any man, especially any young man, who starts out in life with the conviction that money can be better made than by earning it, is a lost man, — lost already to society, lost to his family, lost to himself.

An alarmingly large number of the sons of the rich men of New York are at this moment helpless drunkards. Young men are they, many of them of education, of manly qualities, of generous natures, honorable and high-minded; but the demon of drink has taken such possession of them that a breaking father's heart, a mother's tears and sister's agony avail not to draw them from their deep damnation. Elegant leisure was their ruin.

The best way to save a child from ruin is to bring him up to

"HELP FATHER."

Make children feel that they must do something to support the family, to help along; then two feelings arise which are their salvation, — those of affection and pride; for we naturally love those whom we help, or those whom we struggle together with for a desired object, and nothing so improves a child as to make him feel that he is of some consequence, that he is needed, that he can do something, and that what he does is of worth, is appreciated. William B. Astor made his son John his helper in his office from early years; Cornelius Vanderbilt taught "Billy," as he calls his oldest son, to help him when quite a youth, and later on learned to lean on him, advised with him, consulted him, made him feel that he was a man; and the result is that New York has no more creditable, effective, and substantial citizens than John Jacob Astor and William Vanderbilt.

In all this great metropolis of a continent there is not a more universally thrifty class than the German population, nor any more worthy of respect; they are quiet, kind, industrious, and economical; and there are a thousand Dutchmen to-day who came to New York less than twenty years ago with only the clothes on their backs and the scantiest of purses, but now own a "corner" store, are out of debt, and have an account at the "Spar Bank;" literally the "pile" made up of what money they can "spare" from their business. The trait universal in German families is to put their children, in their earliest "teens," where they can earn something, be it ever so little, to help at home. American parents, think of this, and save your sons from the asylum and your daughters from the street.

